# Study on the Antinociceptive Effects of Herba Epimedium in Mice

**DOI:** 10.1155/2015/483942

**Published:** 2015-06-11

**Authors:** Jiang-hong Sun, Xin-jie Ruan, Li-na Wang, Shuang Liang, Xin-ping Li

**Affiliations:** ^1^Department of Animal Biotechnology, College of Veterinary Medicine, Northwest A & F University, Yangling 712100, China; ^2^Henan Soar Veterinary Pharmaceutical Co., Ltd., Zhengzhou, Henan 45008, China

## Abstract

The present study was conducted to investigate the antinociceptive action of relationship between Herba Epimedium (HE) and 5-HT_1A_ receptor, between Herba Epimedium (HE) and 5-HT_2A_ receptor. We used the hot-plate method and the writhing assay in mice by the intracerebroventricular (i.c.v.) injection and observed the analgesic effect of HE. Furthermore, through the i.c.v. injection, 5-HT_1A_ receptor partial agonist Buspirone, antagonist Propranolol, the adrenaline *β*_1_-receptor selective blocking agent Metoprolol, and 5-HT_2A_ receptor agonist hydrochloride DOI and antagonist Ketanserin were used, and, 5 min later, HE was used to investigate the impacts of drugs on the analgesic effect in the same way. Results showed that HE had fast and significant antinociception in nervous system, and the effects can persist for a long time. Buspirone and Hydrochloride DOI can remarkably increase the antinociception of HE in nervous system. Ketanserin leads to a significant decrease in its antinociception in nervous system; Metoprolol also has antinociceptive action in nervous system, but it can inhibit the antinociceptive effect of Herba Epimediumin peripheral region. These results suggest that HE has significant antinociception effect and its mechanism is related with 5-HT_1A_ receptor, 5-HT_2A_ receptor, and adrenaline *β*_1_-receptor.

## 1. Introduction

The 5-hydroxytryptamine (5-HT) plays an important role in the nociceptive transmission. The involvement of central monoaminergic pathways in the antinociceptive mechanisms has been emphasized, and the role of serotonin (5-HT) in the modulation of the nociceptive stimuli has also been demonstrated [1, 2]. The central serotonergic pathways have been claimed to exert antinociceptive effects in defined brain areas through their receptors, notably 5-HT_1A_ and 5-HT_2A_, as well as through interactions with opioid and *γ*-aminobutyric acid pathways [3–7].

Many previous studies have shown that activation of 5-HT_1A_ receptor can produce a strong analgesic effect [8–10] in the model of formalin-induced chronic pain, surgery, and postoperative pain of neuropathic pain models.

The distribution of 5-HT_2A_ receptor-labelled neurons in dorsal root ganglia (DRG) neurons is mainly small and medium-sized cells, which are nociceptive stimulation of primary sensory neurons. These cells mostly express both pain-related and calcitonin gene-related peptides [11]. The previous researches demonstrated that the antinociceptive effect of 5-HT_2A_ receptor agonist Hydrochloride DOI in the formalin test is dose-dependent in rats [12].

Herba Epimedium (HE) is a family of plants consisting of Epimedium genera, and also is one of the most frequently used herbs in formulas for the treatment of lots of diseases in China. Modern medical researches have showed that HE contains flavonoid compounds and has the antinociceptive effect [13]. HE has the analgesic effect, but there are few researches about it and the detailed mechanisms underlying the analgesic effects of it are still unclear. In the present study, we investigated the mechanism of its analgesic effect using the hot-plate test and the writhing assay, by i.c.v. injections of 5-HT_1A_ receptor, 5-HT_2A_ receptor agonists, and antagonists, with the aim to reveal the relationship of analgesic effect between HE and the 5-HT receptors.

## 2. Materials and Methods

### 2.1. Animals

Adult female Kunming mice were obtained from Animal Center of Medicine Department in Xi'an Jiaotong University. All mice were housed in standard cages and maintained on a 12:12 h light/dark cycle under conditions of constant 23°C ambient temperature. The mice were given a free access to food and water. After one-week acclimatization, these mice were assigned randomly into a group fed with standard mice chow (*n* = 8, weight: 18–22 g).

### 2.2. Drugs

Herba Epimedium (Epimedium enshiense B. L. Guo et Hsiao), the 5-HT_1A_ receptor partial agonist Buspirone, the beta-noradrenergic receptors nonselective antagonist propranolol, the *β*_1_-adrenergic selective antagonist metoprolol, and the 5-HT_2A_ receptor preferential antagonist Ketanserin were purchased from Research Biochemicals. The 5-HT_2A_ receptor selective agonist DOI-Hydrochloride was purchased from Sigma.

### 2.3. Drug Treated Groups

In this study, the mice were divided into the control group, of which each group was treated with i.c.v. injection of saline solution (2 *μ*m/L), the HE + Metoprolol group, of which the mice were treated with Herba Epimedium (1 : 4), the *β*_1_-adrenergic selective antagonist Metoprolol (2 *μ*m/L), and Herba Epimedium, respectively, and four separate groups of mice were treated with the 5-HT_1A_ receptor partical agonist Buspirone (2 *μ*m/L), the beta-noradrenergic receptors nonselective antagonist propranolol (2 *μ*m/L), the 5-HT_2A_ receptor preferential antagonist Ketanserin (1 *μ*m/L), and the 5-HT_2A_ receptor selective agonist DOI-Hydrochloride (1 *μ*m/L), respectively, administered 5 min after Herba Epimedium (1 : 4) administration.

### 2.4. Preparation of Aqueous Extract of Epimedium

Herba Epimedium (100 g) were soaked in (how much) mL of distilled water for an hour and then boiled three times, every time for 30 min. Then the filtration was combined, and the first extraction was concentrated to 200 mL by simmering and then was extracted with 200 mL of 95% ethanol for 3 times and placed at 4°C or −20°C overnight. The compound was filtered. After filtration, each residual was discarded and the final filtrates were concentrated under vacuum to eliminate solvent and double distilled water was added into the extract to the final volume of 10 mL. 2% NaOH was used to adjust the pH to 7.0. The concentration of the herbal soup is equivalent to crude herb of 1 g/mL, kept in refrigerator.

### 2.5. Drugs Administration and i.c.v. Injection

The 5-HT receptors agonists and antagonists were dissolved in sterile 0.9% NaCl as vehicle containing 0.1% Evans Blue dye. This saline solution was used as a vehicle and the control treatment. i.c.v. injection was performed according to a method reported previously [14, 15]. The needle was placed vertically on the head, the needle was lightly forward to locate bregma, and the needle position was 1 mm posterior and 1 mm lateral to the bregma. Herbal solutions were infused slowly (over 30 s) into the lateral ventricle and injected i.c.v. in a volume of 10 *μ*L. At the end of each experiment, the mice were sacrificed by injection of an overdose of urethane. Then the brain was removed to confirm the accuracy of injection. The data from brains which confirmed having Evans Blue dye present in the lateral ventricle were used.

### 2.6. Hot-Plate Test

In this test, animals were individually placed on a hot plate with constant temperature (55 ± 0.5°C) [16]. The latencies to first hind paw withdrawal during thermal stimulation were measured in seconds as indexes of nociceptive threshold with cut-off time of 50-second reaction times were measured with a stopwatch and each of the mice was tested before treatment and 5, 15, and 30 min after treatment. Those mice scores below 10 s or over 60 s in the pretest were rejected, after which the animals were immediately removed from the hot plate.

### 2.7. Writhing Test

The writhing test described by Hayashi and Takemori [17] was used to assess the analgesia effect. Mice were i.c.v. injected with 2% acetic acid in order to produce the typical writhing reaction, which is characterized by a wave of contraction of the abdominal musculature followed by extension of the hind limbs. The mice were then placed in an individual container and the number of writhes in 35 min was counted, starting from 5 min after acetic acid administration.

### 2.8. Statistical Analysis

Data from experiments were statistically analyzed with one-way analysis of variance (ANOVA) and *t*-test. With respect to treatment and time, the Kruskal-Wallis test was used. Data are expressed as means ± SEM. Statistical significance was set at *P* < 0.05 for all experiments.

## 3. Results

### 3.1. The Pain Threshold in Hot-Plate Test of HE + Metoprolol Groups

**Figure 1:**
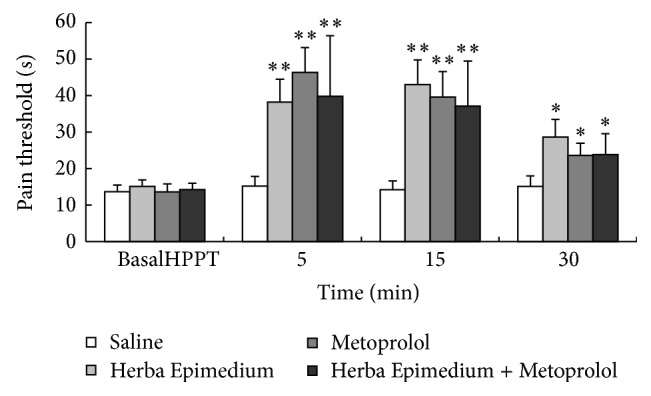
The pain threshold in hot-plate test during 30 min after i.c.v. injection of Herba Epimedium and Metoprolol. Values are means ± S.E.M. Significant difference from the control (saline) at each time point represented ^∗∗^ *P* < 0.01 and ^∗^ *P* < 0.05.

The i.c.v. administration of HE and Metoprolol potently increased the pain threshold 30 min after injection in the mouse hot plate. Antinociception of the Herba Epimedium group reached the maximum after 15 min, and the Metoprolol (2 *μ*mol per mouse) reached the maximum after 5 min. Both of them can persist for up to 30 min. The mice having received i.c.v. HE and Metoprolol exhibited significant antinociception. There was no significant difference in the increase in pain threshold following i.c.v. injection between the HE group and Metoprolol group (*P* > 0.05) (Figure 1).

### 3.2. The Antinociceptive Effect of HE and Metoprolol on the Abdominal Constriction Test

**Figure 2:**
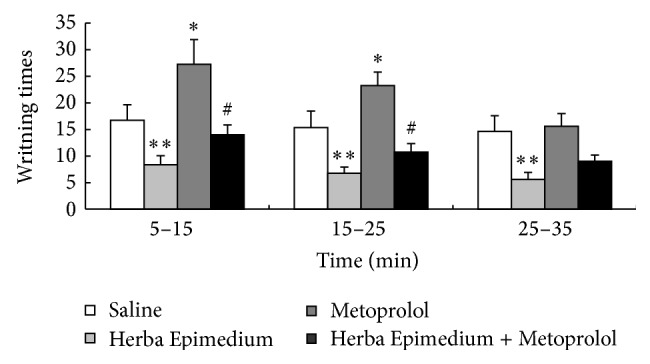
The writhing times in the mouse acetic acid abdominal constriction test during 35 min after i.c.v. injection of HE and Metoprolol. Values are means ± S.E.M. Significant difference from the control (saline) at each time point represented ^∗∗^ *P* < 0.01 and ^∗^ *P* < 0.05.  ^#^ *P* < 0.05 compared to HE.

The antinociceptive effects of Herba Epimedium and Metoprolol on the abdominal constriction test were illustrated in Figure 2. The abdominal constriction test was performed 35 min after i.c.v. administration. The writhing times of mouse were significantly decreased by the HE (*P* < 0.01) and persisted for up to 35 min (*P* < 0.05), but the effect of Metoprolol was different from those in the hot-plate test. It had no antinociceptive effect compared to the vehicle group. The writhing times were significantly increased during 25 min after the administration (^∗^ *P* < 0.05). Posttreatment after 35 min had no significant difference from the control group. Whereas the writhing time was significantly decreased after injection of HE and Metoprolol compared to the Metoprolol group during 25 min after the administration (^#^ *P* < 0.05), the magnitude of the three phase did not differ significantly from the Metoprolol group.

### 3.3. The Effect of i.c.v. Injection of HE, Buspirone, and Propranolol

**Figure 3:**
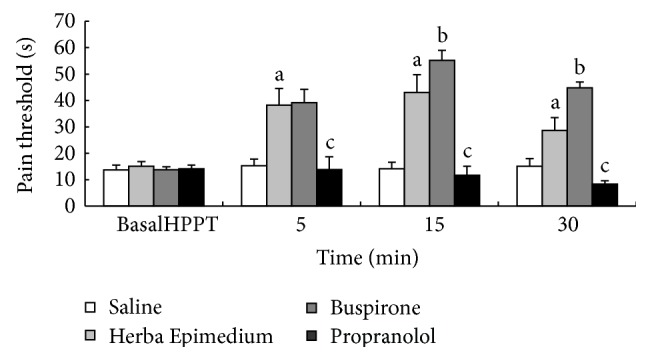
Pain threshold in hot-plate test during 30 min after i.c.v. injection of HE, Buspirone, and Propranolol. Values are means ± S.E.M. ^a^ *P* < 0.01 compared to corresponding control group. ^b^ *P* < 0.05, ^c^ *P* < 0.01 compared to corresponding Herba Epimedium group.

The effect of i.c.v. injection of HE, Buspirone, and Propranolol on 30 min pain threshold value was shown in Figure 3. In the hot-plate test, HE strongly increased the pain threshold value and persisted for up to 30 min (^a^ *P* < 0.01); 5-HT_1A_ receptor partial agonist Buspirone can increase the antinociceptive effect of HE 15 min after injection (^b^ *P* < 0.05). However, the antagonist Propranolol significantly decreased the antinociception of HE through 30 min after injection (^c^ *P* < 0.01).

### 3.4. The Effect of 5-HT_2A_ Receptor Agonist (±)-DOI Hydrochloride and Antagonist Ketanserin

**Figure 4:**
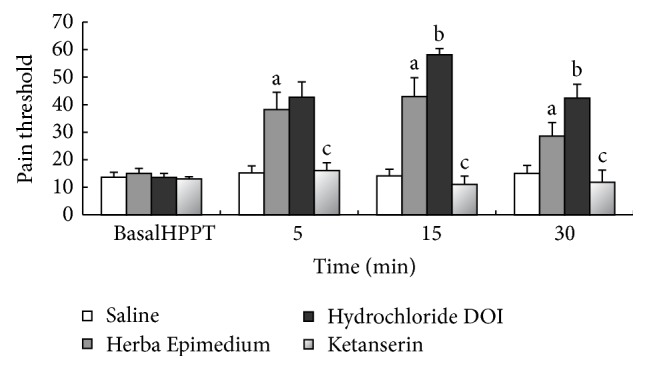
Pain threshold in hot-plate test during 30 min after i.c.v. injection of HE, Hydrochloride DOI, and Ketanserin. Values are means ± S.E.M. ^a^ *P* < 0.01 compared to corresponding control group. ^b^ *P* < 0.05, ^c^ *P* < 0.01 compared to corresponding HE group.

The i.c.v. administration of 5-HT_2A_ receptor agonist (±)-DOI Hydrochloride increases the antinociceptive effect of HE at 15 min and 30 min (^b^ *P* < 0.05) in the hot-plate test, but the 5-HT_2A_ receptor antagonist Ketanserin inhibited the antinociception of HE at 30 min after injection (^c^ *P* < 0.01) (Figure 4).

### 3.5. The Antinociception of HE, Propranolol, and Buspirone in the Acetic Acid Abdominal Constriction Test

**Figure 5:**
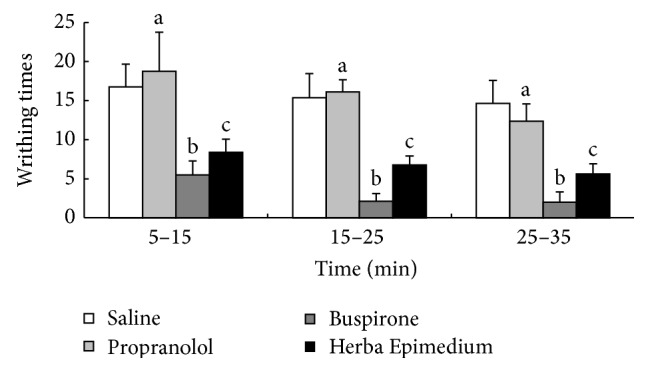
The writhing times in the mouse acetic acid abdominal constriction test during 35 min after i.c.v. injection of HE, Propranolol, and Buspirone. Values are means ± S.E.M. ^a^ *P* < 0.01, ^b^ *P* < 0.05 compared to corresponding Herba Epimedium group. ^c^ *P* < 0.01 compared to corresponding control group.

This experiment indicated that the 5-HT_1A_ receptor antagonist Propranolol can increase the writhing times in the test compared to the control group and the magnitude of inhibitory effect was similar to that experiment of hot plate. 5-HT_1A_ receptor partial agonist Buspirone can also increase the antinociception of HE in the acetic acid abdominal constriction test. The writhing times significantly decreased compared with the HE group (^b^ *P* < 0.05) (Figure 5).

### 3.6. The Antinociceptive Effects of Hydrochloride DOI and Ketanserin in the Acetic Acid Abdominal Constriction Test

**Figure 6:**
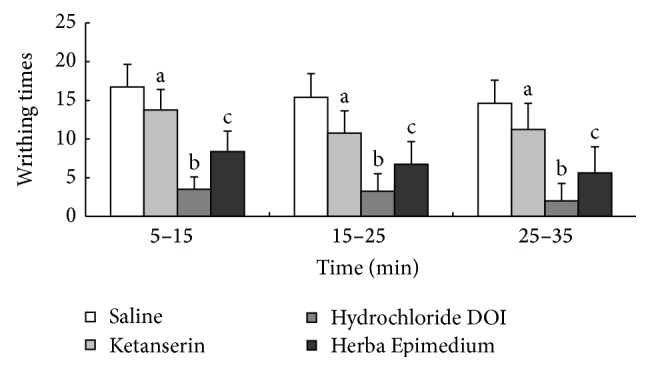
The writhing times in the mouse acetic acid abdominal constriction test during 35 min after i.c.v. injection of HE, Hydrochloride DOI, and Ketanserin. Values are means ± S.E.M. ^a^ *P* < 0.05, ^b^ *P* < 0.05 compared to corresponding HE group. ^c^ *P* < 0.01 compared to corresponding control group.

The abdominal constriction test was performed 35 min after i.c.v. administration. The test indicated that 5-HT_2A_ receptor antagonist Ketanserin could suppress the antinociception of HE; the writhing times of mouse were significantly increased at 35 min after injection, compared to the HE group (^a^ *P* < 0.05). 5-HT_2A_ receptor agonist (±)-DOI Hydrochloride could increase the antinociceptive effect of HE and the writhing times decreased 35 min after injection (^b^ *P* < 0.05) (Figure 6).

## 4. Discussions

Herba Epimedium, which contains several medically active constituents including flavonoids and phytosteroids, has been widely used in China in the treatment of cardiovascular diseases, infertility, impotence, amnesia, lumbago, arthritis, numbness, and weakness of the limbs for thousands of years [18, 19]. Recently, several studies have shown that the crude extract, total flavonoids, and main flavonoid constituents from Herba Epimedium had the antinociception effect [13, 20]. In our study, the Herba epimedium significantly elevated the pain threshold in the mouse hot-plate (Figure 1) and abdominal constriction (Figure 2) tests. Total flavonoids and main flavonoid constituents had the antinociceptive effect [21]. However, the mechanism of antinociception of Herba Epimedium aqueous extract which contained flavonoid was not clear. Adrenaline *β*_1_-receptor selective blocking agent has sedative, analgesic, and anxiolytic effects by blocking the central *β* receptor [22]. In the present study, Metoprolol showed opposite effects between the hot-plate tests (Figure 1). And in abdominal constriction test (Figure 2), Metoprolol has the significant antinociception in the central nervous system, but has the role of pain in peripheral region. It was possible that Metoprolol only had the antinociceptive effect in central nervous system. The increase of pain threshold was also detected by injection of both Metoprolol and Herba Epimedium, the results indicated that Herba Epimedium had the inhibitory effect to the Metoprolol, especially in the abdominal constriction test, and the writhing times of mouse were significantly decreased 35 min after injection (*P* < 0.01) in the present study. In vitro and animal experiment had proved that total flavones of Herba Epimedium have the selective block effect on adrenergic *β*_1_ receptor [23].

5-HT is an endogenous bioactive substance released from platelets and mast cells in injured or inflamed tissues. Endogenous or exogenous 5-HT causes noxious and hyperalgesic reactions in peripheral tissues in human and animals. Intracutaneous injection of platelets causes acute pain and hyperalgesia in human [24], it was reported that subtly 5-HT receptors were involved in 5-HT induced pain and hyperalgesia. So many selective drugs have been allowed to develop for treating inflammatory pain and hyperalgesia. Bianchi et al. [25] demonstrated that the full 5-HT_1A_ agonist 8-OH-DPAT was able to increase ACh release from the cerebral cortex of freely moving guinea pigs. 5-HT_1A_ receptor partial agonist Buspirone was able to induce antinociception in mice; the increased pain threshold was detected by using both thermal (hot-plate test) and chemical stimuli (abdominal constriction test) [26]. In the present study, the result indicated that Buspirone can significantly increase the antinociceptive effect of Herba Epimedium; it was possible that analgesic of Herba Epimedium was related to the 5-HT_1A_ receptor. The antinociception of Herba Epimedium was suppressed by the antagonist Propranolol.

It is well known that 5-HT is involved in many complex effects on pain and hyperalgesia through various subtype of receptors located at various levels of the pain transmission system. In a previous experiment, the intraperitoneal administration of acetic acid formalin evoked extremely long-lasting inhibition of somatic inflammatory pain in mice. The study indicated that long-lasting antinociception was completely blocked by the 5-HT_2A_ receptor antagonists, Ketanserin [27]. Peiró et al. reported 5-HT inhibition of the kappa-opioid component of morphine antinociceptive effects and the possibility that this serotonergic inhibition could be reversed through 5-HT2 receptor antagonist Ketanserin [28]. Crisp et al. reported that intrathecal administration of 5-HT agonist, 8-OH-DPAT produced hyperalgesia with the tail-flick test and analgesia with the hot-plate test, and 5-HT_1B_ agonist, TFMPP, 5-HT_2A_ agonist and DOI phenylbiguanide produced analgesia with the hot-plate test in the rat [29]. Therefore, the 5-HT receptor agonists or antagonists may act and change the pain-related behavior. In this study, the i.c.v. administration of 5-HT_2A_ receptor agonist (±)-DOI Hydrochloride can increase the antinociceptive effect of Herba Epimedium at 15 min and 30 min (*P* < 0.05) in the hot-plate test, but the 5-HT_2A_ receptor antagonist Ketanserin inhibited the antinociception of Herba Epimedium through 30 min after injection (*P* < 0.01). As reported previously [30], the Ketanserin has a nonselective action, also exerting some effects on alpha-1-noradrenergic receptors.

In the acetic acid abdominal constriction test, the study indicated that 5-HT_2A_ receptor antagonist Ketanserin could suppress the antinociception of Herba Epimedium. The writhing times of mouse were significantly increased through 35 min after injection, which compared to the Herba Epimedium group (^a^ *P* < 0.05). 5-HT_2A_ receptor agonist (±)-DOI Hydrochloride could increase the antinociceptive effect of Herba Epimedium and the writhing times decreased during the 35 min (^b^ *P* < 0.05). Therefore, the hyperalgesia induced by the hot-plate test and acetic acid test was inhibited by Herba Epimedium, and the antinociception of Herba Epimedium was mediated via the 5-HT_2A_ receptor agonist and antagonist.

Several evidences indicated that 5-HT_1A_ and 5-HT_2A_ receptor subtype contributed to antinociception. Moreover, Herba Epimedium has the antinociceptive effect in central nervous system and peripheral region [13, 20]. At the moment, we speculate that the antinociception of Herba Epimedium was mediated by the agonist and antagonist of 5-HT_1A_ receptor and 5-HT_2A_ receptor, but the mediating mechanisms are not clear; further studies are necessary to characterize this pain facilitatory mechanism.

## 5. Conclusions

From all above experiments, we concluded that HE had significant antinociceptive effect and the mechanism of its antinociception was connected with 5-HT_1A_ receptor, 5-HT_2A_ receptor, and adrenaline *β*_1_-receptor.
